# Curvature-Waviness Interaction in Self-Locking Honeycombs: Contact-Driven Compression and Progressive Compaction

**DOI:** 10.3390/ma19194198

**Published:** 2026-09-30

**Authors:** Abir Rouis, Zongbing Chen, Chengrui Yan, Cherif Othmani, Jian Xiong

**Affiliations:** 1 National Key Laboratory of Science and Technology for Advanced Composites in Special Environments, Center for Composite Materials and Structures, Harbin Institute of Technology, Harbin 150080, China; rouis.abir@hit.edu.cn (A.R.); zongbingchen@126.com (Z.C.); 23s018036@stu.hit.edu.cn (C.Y.); 2 Structural Lightweighting and Composites Laboratory (StruCM Lab), Center for Composite Materials and Structures, Harbin Institute of Technology, Harbin 150080, China; 3 Laboratory of Measurement and Sensor System Techniques, TUD Dresden University of Technology, 01069 Dresden, Germany; cherif.othmani@tu-dresden.de

**Keywords:** lightweight structures, self-locking structure, curved cellular structure, contact morphology, energy absorption

## Abstract

Self-locking honeycombs exploit intercellular contact to regulate deformation and load transfer during compression, yet the coupled effects of wall curvature and 3D contact morphology remain insufficiently understood. The present work develops a two-parameter geometric framework in which the in-plane curvature angle (α) and through-height waviness index (m) independently control the morphology of curved walls that simultaneously carry load and provide intercellular contact. Twelve additively manufactured PA12 self-locking assemblies were investigated by frameless quasi-static compression and nonlinear finite-element simulations. Increasing wall waviness generally enhanced initial stiffness and peak resistance, whereas the influence of curvature angle was non-monotonic. The configuration with m=3, α=60° achieved the highest mean mass-normalized peak force of 17.11 N/g, while m=3, α=45° provided the highest mean specific energy absorption of 0.59 J/g and crush force efficiency of 0.78 at 45% strain. Deformation observations and simulations indicate that stable progressive compaction requires sufficient intercellular engagement while preserving the sliding, rotation, recontact, and compatible void closure needed for continued deformation. Excessive geometric constraint can instead promote row misalignment and interrupted compaction. The framework provides a design basis for lightweight energy-absorbing cores in protective panels, transportation crash management structures, and packaging systems, subject to future dynamic validation.

## 1. Introduction

Honeycombs are widely used in lightweight load-bearing and energy absorption (EA) applications because their mechanical response can be tailored through geometry as well as material selection [1,2,3,4,5]. Classical hexagonal honeycombs are commonly interpreted through relative density and cell wall bending, stretching, buckling, and nodal rotation [3,6,7]. With additive manufacturing, however, relatively thick walls, strong curvature, and three-dimensional surface features can be introduced deliberately, expanding the design space beyond the assumptions of conventional slender-wall descriptions [1,5,8]. Recent studies on additive manufactured cellular structures show that geometric modification such as progressive wall-thickness variation, hybrid cell layout, and hierarchical features can substantially alter compressive deformation and EA performance [9,10,11,12]. For such architectures, wall geometry can influence not only structural deformation but also the interaction between neighboring cells during large compression.

Curved cellular members have attracted increasing attention as mechanical design variables. Harkati et al. [13] showed that replacing straight honeycomb walls with curved walls alters the in-plane elastic constants, Poisson’s ratio, and shear response. Álvarez-Trejo et al. [14], Dong et al. [15], and Farrokhabadi and Chronopoulos [16] further demonstrated that mathematically controlled curved members can tailor stiffness, deformation mode, auxetic response, crushing resistance, and EA. More recently, several studies [17,18,19] investigated geometrically modified auxetic honeycombs and demonstrated that controlled cell wall geometry can alter deformation behavior, in-plane mechanical response, crushing performance, and EA. Zhang et al. [20] further showed that variation of the cell rotation angle influences deformation mode, SEA, and crush force efficiency (CFE). A related body of research concerns topological interlocking, in which structural integrity arises from geometric constraints and contact between discrete elements. Dyskin et al. [21] established the fundamental concept, while Siegmund et al. [22] summarized its manufacturing and mechanical principles. Djumas et al. [23] investigated hierarchical non-planar interlocked assemblies, whereas Weizmann et al. [24] and Kim and Siegmund [25] demonstrated the influence of block and tessellation geometry on structural behavior. Mirkhalaf et al. [26] showed that interlocking can simultaneously improve strength and toughness. More recently, Lu et al. [27] demonstrated the roles of friction and geometry in interlocking stiffness, while Koureas et al. [28,29] investigated failure and hierarchical interlocking. Dalaq and Barthelat [30] further showed that interface geometry can be tailored to control strength and toughness.

Self-locking energy absorbers exploit related geometric interactions to provide mutual restraint between neighboring components during compression. Chen et al. [31] introduced a self-locked energy-absorbing system, and Yang et al. [32] subsequently demonstrated that element shape and geometry can regulate its deformation and EA. Zhao et al. [33] extended the concept to bidirectional self-locking under multiple compression directions. Qin et al. [34] developed a node-locked multicell honeycomb for efficient EA, while Niu et al. [35] investigated the in-plane compression behavior of a self-locked chiral honeycomb. Gao and You [36] introduced self-locking into Miura Ori honeycombs, Chen et al. [37] developed an origami-based bidirectional self-locking system, and Zhu et al. [38] investigated an auxetic self-locking architecture for improved crashworthiness.

Despite these developments, most curved and self-locking architectures are investigated as predefined geometric configurations, making it difficult to isolate the effects of individual morphological variables. Recent mathematical descriptions of curved interlocking interfaces, such as that proposed by Tavoosi Gazkoh et al. [39], demonstrate the value of explicit geometric parameterization. A broader trend toward systematic parameterization is also evident in architected materials and mechanical metamaterials. Yin et al. [40] developed generic design criteria based on cell properties and spatial arrangement to obtain programmable mechanical properties, while Mandolesi et al. [41] used two dimensionless parameters to tailor the homogenized elastic response of spinodal-inspired metamaterials. Although these studies concern different architectures, they demonstrate the broader value of explicit design parameters for relating structural geometry to mechanical response. In our previous study [42], SSC, CSC, SDC, and internally reinforced arc-curved self-locking honeycombs were investigated experimentally and numerically under frame-confined compression, demonstrating that contact geometry and interface conditions influence peak resistance, EA, and deformation behavior. However, wall curvature and contact wall morphology were not varied independently. Moreover, the imposed confinement limited direct assessment of the sliding, rotation, recontact, and row rearrangement that can develop during unrestricted compaction. These limitations motivate a controlled parametric approach in which the two geometric features are varied independently within the same parent architecture and evaluated under frameless compression. Beyond this methodological distinction, the present study addresses the physical balance between intercellular engagement and deformation compatibility. The frameless configuration allows the relative motions suppressed by lateral confinement to develop, making it possible to determine whether stronger geometric engagement consistently improves compression behavior or instead becomes detrimental once sliding, rotation, recontact, and compatible row rearrangement are excessively restricted. The resulting comparison shows that early load resistance and stable progressive compaction are distinct consequences of contact morphology rather than necessarily coincident design objectives.

The present study therefore develops a two-parameter geometric framework to determine how curvature angle and contact wall waviness affect the compressive performance of arc-curved self-locking honeycombs. The architecture is first constructed through computer-aided design (CAD) using profile rotation and lofting and is subsequently described mathematically using six piecewise boundary functions and a height-dependent surface formulation. The curvature angle (α) controls the in-plane wall curvature while maintaining a constant parent cell chord length, whereas the waviness index (m) controls the through-height contact morphology. Four curvature angles and three surface morphologies generate twelve configurations. Additively manufactured PA12 assemblies are investigated through frameless quasi-static compression and nonlinear finite-element (FE) simulations to evaluate initial stiffness, peak resistance, EA, CFE, deformation mode, and progressive compaction. This framework enables the effects of curvature and waviness to be examined systematically within a common self-locking architecture.

## 2. Parametric Geometry and Design Space

The self-locking architecture studied is derived from a hexagonal unit cell with a circular arc, composed of six load-bearing walls which, after assembly, form the inter-cell contact surfaces. Two complementary approaches are used to define the geometry. The first is CAD modeling, used to generate the physical specimens. The second is a mathematical parameterization developed to systematically reproduce the geometric construction and independently prescribe the in-plane curvature and the vertical contact morphology.

**Figure 1:**
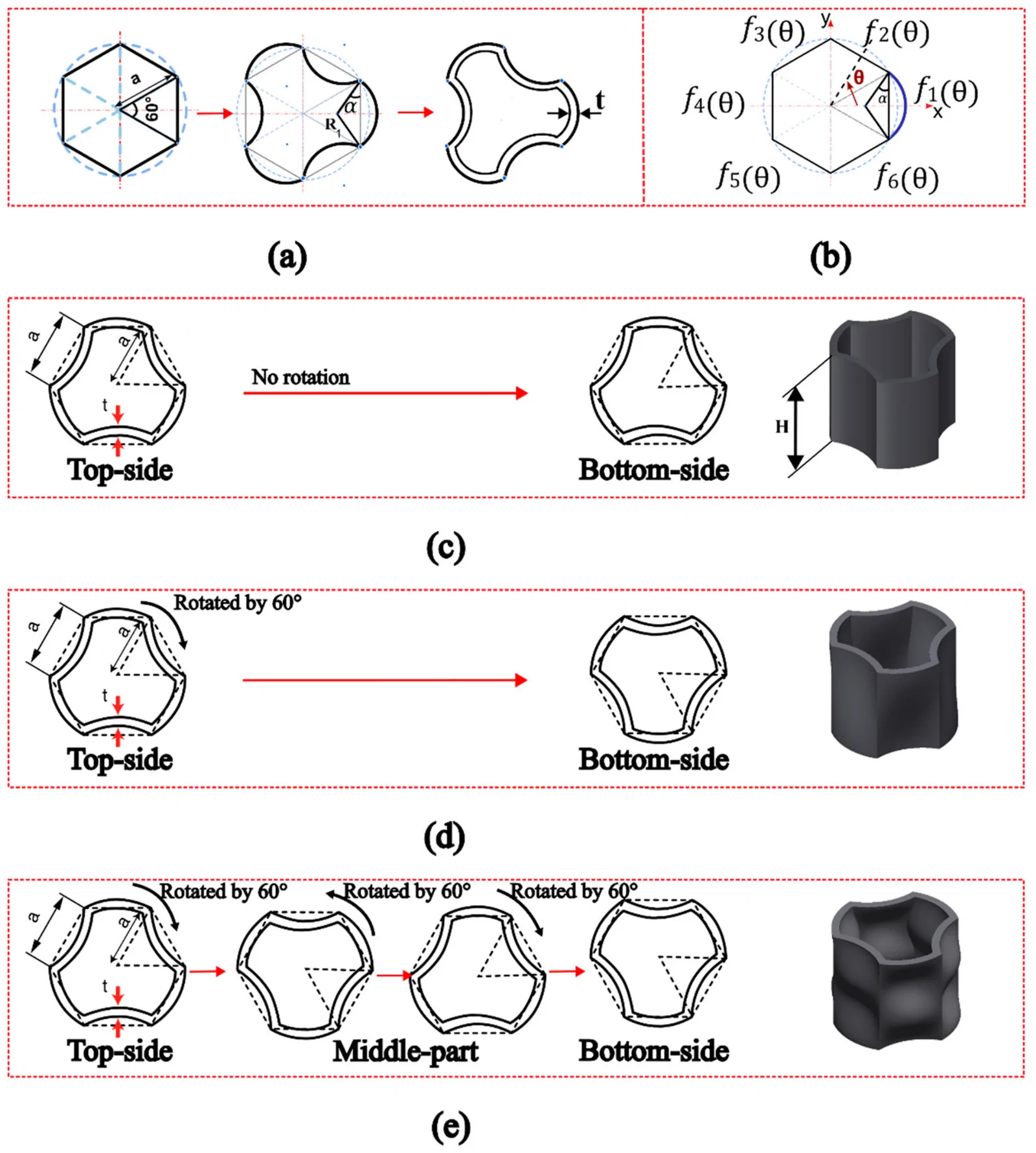
Parametric geometric construction of the arc-curved self-locking honeycomb. (**a**) Definition of curvature angle α and arc radius $R_{1}$, with constant chord length a and thickness t. (**b**) Planar unit-cell profile defined by six curve segments $f_{1}\left(\theta \right),\;f_{2}(\theta )$, $f_{3}(\theta )$, $f_{4}(\theta )$, $f_{5}(\theta )$, and $f_{6}(\theta )$ arranged counterclockwise on the parent hexagonal topology. (**c**) SFS configuration (m=0) extruded without through-height rotation. (**d**) SSC configuration (m=1) generated by 60° rotation between top and bottom profiles. (**e**) SDC configuration (m=3) generated by successive 60° rotations between four profiles. Wall cell height H.

In the CAD route, all configurations originate from the same hexagonal base and differ in the profile arrangement used to generate the contact wall. The simple flat structure SFS configuration contains no through-height profile rotation and therefore forms a flat extruded contact surface, as shown in Figure 1c. For simple single-contact SSC, the top and bottom profiles are rotated by 60° relative to one another and connected by lofting, producing a single curved contact morphology across the cell height, as shown in Figure 1d. For simple double-contact SDC, four profiles (top, middle 1, middle 2, and bottom) are used, with each adjacent profile rotated by 60° before lofting, as shown in Figure 1e. This successive rotation produces the more strongly varying SDC contact surface. The same construction principle is subsequently expressed mathematically to permit systematic generation of the complete design space without independently reconstructing each configuration in CAD.

The reference chord length is a=12 mm, the wall thickness is t=1.5 mm, and the cell height is H=19.596 mm, corresponding to H=1.633a. The relative wall thickness is therefore t/a=0.125. Two morphological parameters are varied: the curvature angle α, which controls the in-plane curvature of the cell wall, and the dimensionless waviness index m, which controls the through-height variation of the contact surface. The investigated design space comprises α=30°, 45°, 60°, and 75°, combined with m=0, 1, and 3. The α=60° case corresponds to the reference curvature setting inherited from the regular hexagonal construction, while α=30° case was adopted as the lowest curvature angle in the present design matrix, reflecting the practical manufacturability limit encountered under the MJF processing. The intermediate angles provide a systematic variation around the reference geometry.

The selected parameter values represent a controlled sampling of the (α,m) design space rather than an exhaustive parametric sweep. The waviness parameter m is not intrinsically restricted to the experimentally investigated values, and intermediate values such as m=2 are mathematically admissible within the present formulation. For the experimental matrix m=0, 1, and 3 were selected to represent three distinctly separated through-height contact morphologies: the flat SFS reference, the lower-waviness SSC morphology, and the higher-waviness SDC morphology, respectively. Thus, the omission of m=2 does not indicate a geometric or manufacturing prohibition; it represents an experimentally untested intermediate state within the continuous parameterized design space.

For the curvature parameter, α=60° was retained as the reference configuration adopted in our previous work, while α=30°, 45°, and 75° were selected to provide lower and higher curvature variations around this reference while preserving the same parent chord length. These values were not obtained from a preliminary optimization.

### 2.1. Two-Dimensional Outer Boundary

The planar unit-cell profile is defined in the x−y plane by six continuous curve segments,

(1) $$f_{i}\left(\theta \right):\left\{\begin{array}{l} X_{i}=X_{i}\left(\theta \right),\\ Y_{i}=Y_{i}\left(\theta \right), \end{array}\right.\;i=1,2,\ldots ,6$$

where θ is the local angular coordinate. The six segments correspond to the six sides of the parent hexagonal topology and are arranged counterclockwise around the cell center. Segment $f_{1}(\theta )$ defines the right-hand curved wall, while $f_{2}(\theta )$, $f_{3}(\theta )$, $f_{4}(\theta )$, $f_{5}(\theta )$, and $f_{6}(\theta )$ reproduce the remaining sides through the same rotational and translational construction. The geometrical construction and angular definitions are shown in Figure 1a,b.

The curvature of each wall is controlled by the construction angle

(2) $$\phi_{1}=180-2\alpha$$

and the corresponding arc radius

(3) $$R_{1}=\frac{a/2}{sin\left(\phi_{1}/2\right)}=\frac{a/2}{cos\left(\alpha \right)}$$

The six functions $f_{1}\left(\theta \right)$–$f_{6}\left(\theta \right)$ are retained as Equations (4a)–(4f). Their prescribed θ-intervals ensure that each function occupies one side of the parent hexagonal layout and that adjacent segments meet at the corresponding vertices.

(4a) $$f_{1}\left\{\begin{array}{c} X_{1}=R_{1}\left[\cos\left(\frac{3\emptyset_{1}}{\pi }\theta \right)-\cos\left(\frac{\emptyset_{1}}{2}\right)\right]+\frac{a\sqrt{3}}{2}\\ Y_{1}=R_{1}\sin\left(\frac{3\emptyset_{1}}{\pi }\theta \right) \end{array}\right.\;\theta \in \left[-\frac{\pi }{6},\frac{\pi }{6}\right]$$

(4b) $$f_{2}\left\{\begin{array}{c} X_{2}=-\frac{R_{1}}{2}\left[\cos\left(\frac{{3\emptyset }_{1}}{\pi }\left(\theta -\frac{\pi }{3}\right)\right)-\cos\left(\frac{\emptyset_{1}}{2}\right)\right]+\frac{a\sqrt{3}}{4}-\frac{\sqrt{3}R_{1}}{2}\sin\left(\frac{{3\emptyset }_{1}}{\pi }\left(\theta -\frac{\pi }{3}\right)\right)\\ Y_{2}=-\frac{\sqrt{3}R_{1}}{2}\left[\cos\left(\frac{{3\emptyset }_{1}}{\pi }\left(\theta -\frac{\pi }{3}\right)\right)-\cos\left(\frac{\emptyset_{1}}{2}\right)\right]+\frac{3a}{4}+\frac{R_{1}}{2}\sin\left(\frac{{3\emptyset }_{1}}{\pi }\left(\theta -\frac{\pi }{3}\right)\right) \end{array}\right.\;\theta \in \left[\frac{\pi }{6},\frac{\pi }{2}\right]$$

(4c) $$f_{3}\left\{\begin{array}{c} X_{3}=-\frac{R_{1}}{2}\left[\cos\left(\frac{{3\emptyset }_{1}}{\pi }\left(\theta -\frac{2\pi }{3}\right)\right)-\cos\left(\frac{\emptyset_{1}}{2}\right)\right]-\frac{a\sqrt{3}}{4}-\frac{\sqrt{3}}{2}R_{1}\sin\left(\frac{{3\emptyset }_{1}}{\pi }\left(\theta -\frac{2\pi }{3}\right)\right)\\ Y_{3}=\frac{\sqrt{3}R_{1}}{2}\left[\cos\left(\frac{{3\emptyset }_{1}}{\pi }\left(\theta -\frac{2\pi }{3}\right)\right)-\cos\left(\frac{\emptyset_{1}}{2}\right)\right]+\frac{3a}{4}-\frac{1}{2}R_{1}\sin\left(\frac{{3\emptyset }_{1}}{\pi }\left(\theta -\frac{2\pi }{3}\right)\right) \end{array}\right.\theta \in \left[\frac{\pi }{2},\frac{5\pi }{6}\right]$$

(4d) $$f_{4}\left\{\begin{array}{c} X_{4}=R_{1}\left[\cos\left(\frac{3\emptyset_{1}}{\pi }(\theta -\pi )\right)-\cos\left(\frac{\emptyset_{1}}{2}\right)\right]-\frac{a\sqrt{3}}{2}\\ Y_{4}=-R_{1}\sin\left(\frac{3\emptyset_{1}}{\pi }(\theta -\pi )\right) \end{array}\right.\;\theta \in \left[\frac{5\pi }{6},\frac{7\pi }{6}\right]$$

(4e) $$f_{5}\left\{\begin{array}{c} X_{5}=-\frac{R_{1}}{2}\left[\cos\left(\frac{{3\emptyset }_{1}}{\pi }\left(\theta -\frac{4\pi }{3}\right)\right)-\cos\left(\frac{\emptyset_{1}}{2}\right)\right]-\frac{a\sqrt{3}}{4}+\frac{\sqrt{3}}{2}R_{1}\sin\left(\frac{{3\emptyset }_{1}}{\pi }\left(\theta -\frac{4\pi }{3}\right)\right)\\ Y_{5}=-\frac{\sqrt{3}R_{1}}{2}\left[\cos\left(\frac{{3\emptyset }_{1}}{\pi }\left(\theta -\frac{4\pi }{3}\right)\right)-\cos\left(\frac{\emptyset_{1}}{2}\right)\right]-\frac{3a}{4}-\frac{1}{2}R_{1}\sin\left(\frac{{3\emptyset }_{1}}{\pi }\left(\theta -\frac{4\pi }{3}\right)\right) \end{array}\right.\theta \in \left[\frac{7\pi }{6},\frac{3\pi }{2}\right]$$

(4f) $$f_{6}\left\{\begin{array}{c} X_{6}=-\frac{R_{1}}{2}\left[\cos\left(\frac{{3\emptyset }_{1}}{\pi }\left(\theta -\frac{5\pi }{3}\right)\right)-\cos\left(\frac{\emptyset_{1}}{2}\right)\right]+\frac{a\sqrt{3}}{4}+\frac{\sqrt{3}}{2}R_{1}\sin\left(\frac{{3\emptyset }_{1}}{\pi }\left(\theta -\frac{5\pi }{3}\right)\right)\\ Y_{6}=\frac{\sqrt{3}R_{1}}{2}\left[\cos\left(\frac{{3\emptyset }_{1}}{\pi }\left(\theta -\frac{5\pi }{3}\right)\right)-\cos\left(\frac{\emptyset_{1}}{2}\right)\right]-\frac{3a}{4}+\frac{1}{2}R_{1}\sin\left(\frac{{3\emptyset }_{1}}{\pi }\left(\theta -\frac{5\pi }{3}\right)\right) \end{array}\right.\;\theta \in \left[\frac{3\pi }{2},\frac{11\pi }{6}\right]$$

Consequently, the complete outer profile can be written as

(5) $$\Gamma_{out}=\underset{i=1}{\overset{6}{⋃}}f_{i}\left(\theta \right)$$

Unlike case-by-case CAD, this formulation provides a reproducible geometrical definition of the complete curved profile and removes the need to reconstruct each curvature case independently from a CAD sketch.

### 2.2. Constant-Thickness Inner Boundary

The structural wall is generated from the outer profile by applying a constant normal offset equal to the prescribed wall thickness t. For each segment, the tangent vector is

(6) $$t_{i}\left(\theta \right)=\frac{dr_{i,out}\left(\theta \right)}{d\theta }$$

and the inward unit normal is defined as

(7) $${\hat{n}}_{i}\left(\theta \right)=\frac{1}{\sqrt{{\left(\frac{dX_{i}}{d\theta }\right)}^{2}+{\left(\frac{dY_{i}}{d\theta }\right)}^{2}}}\left[\begin{array}{c} -\frac{dY_{i}}{d\theta }\\ \frac{dX_{i}}{d\theta } \end{array}\right]$$

The sign of the normal is selected such that it points toward the interior of the cell. The corresponding inner boundary is then obtained from

(8) $$r_{i,in}\left(\theta \right)=r_{i,out}\left(\theta \right)+t{\hat{n}}_{i,in}\left(\theta \right)$$

For the circular contact segment, this operation produces a concentric inner arc with radius $R_{i}=R_{1}-t$. Applying the same offset procedure to all six segments generates a closed inner profile with constant prescribed wall thickness along the complete cell perimeter. This step is important because the present geometry has t/a=0.125; consequently, the wall is represented by its finite thickness rather than by an idealized zero thickness middle surface.

### 2.3. Three-Dimensional Contact-Surface Formulation

After defining the two-dimensional outer and inner profiles, the cell is extended through the height direction z. For a prescribed curvature angle α, the corresponding circular-arc radius is

(9) $$R_{\alpha }=\frac{a/2}{cos\left(\alpha \right)}$$

and the associated sagitta is

(10) $$A_{\alpha }=R_{\alpha }-\sqrt{R_{\alpha }^{2}-{\left(\frac{a}{2}\right)}^{2}}$$

Here $A_{\alpha }$ represents the maximum in-plane deviation of the curved contact wall from the corresponding straight side of the parent hexagon. Increasing α increases $R_{\alpha }$ and decreases the curvature depth $A_{\alpha }$, as shown in Table 1, producing a progressively shallower in-plane wall curvature.

**Figure 2:**
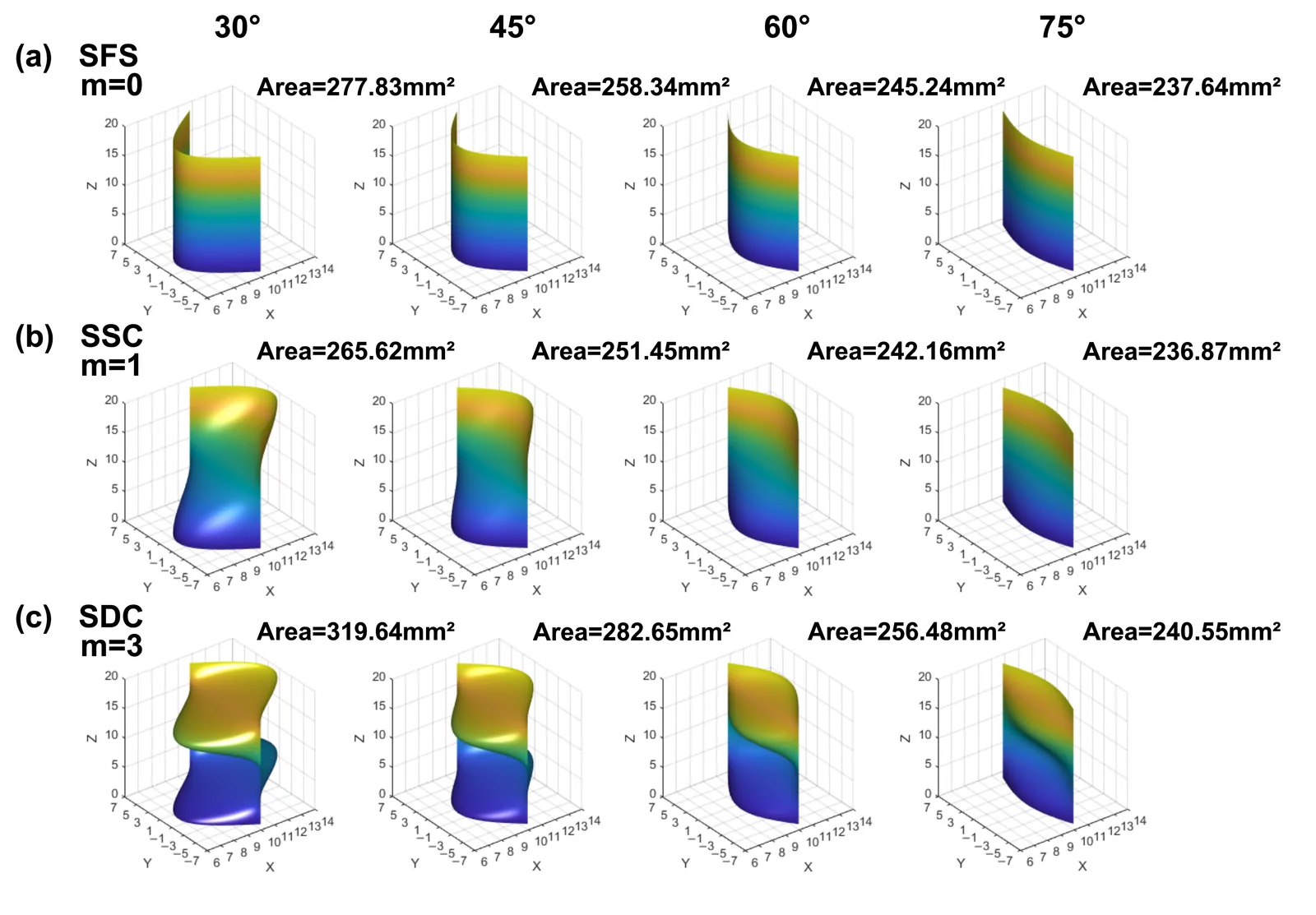
Representative one-sided contact surfaces generated by the parametric formulation for (**a**) SFS (m=0), (**b**) SSC (m=1), (**c**) SDC (m=3) at α = 30°, 45°, 60°, and 75°. The annotated values indicate the corresponding contact surface area $A_{c}$.

Using the local contact coordinate s and the height coordinate z, the waviness-controlled representative surface is defined as

(11) $$\left\{\begin{array}{c} X\left(s,z\right)=C-A_{\alpha }\cos\left(\frac{m\pi z}{H}\right)\cos\left(\frac{\pi s}{a}\right)\\ Y\left(s,z\right)=s\\ Z\left(s,z\right)=z \end{array}\right.$$

where $C=\left(\frac{\sqrt{3}}{2}\right)a$, −a/2≤s≤a/2, and 0≤z≤H. The parameter m controls the through-height variation of the contact morphology. In the investigated design matrix, m=0 defines the SFS family, m=1 defines the SSC family, and m=3 defines the SDC family. Thus, α primarily controls the planar curvature of the wall, whereas m controls the three-dimensional waviness of the contact surface. The resulting contact surfaces for the investigated (α,m) combinations are shown in Figure 2.

For a general two-dimensional boundary segment,

(12) $$r_{i,2D}\left(\theta \right)=\left[\begin{array}{c} X_{i}\left(\theta \right)\\ Y_{i}\left(\theta \right) \end{array}\right],\;i=1,2,\ldots ,6$$

The corresponding three-dimensional surface is expressed as

(13) $$S_{i}\left(\theta ,z\right)=\left[\begin{array}{c} X_{i}\left(\theta \right)+\Delta X_{i}\left(\theta ,z\right)\\ Y_{i}\left(\theta \right)+\Delta Y_{i}\left(\theta ,z\right)\\ z \end{array}\right],\;0\leq z\leq H$$

The terms $\Delta X_{i}\left(\theta ,z\right)$ and $\Delta Y_{i}\left(\theta ,z\right)$ represent the local height-dependent modulation applied along the appropriate normal direction of each boundary segment. For the representative contact side, the modulation reduces to

(14) $$\Delta X\left(s,z\right)=A_{\alpha }cos\left(\frac{m\pi z}{H}\right)cos\left(\frac{\pi s}{a}\right)$$

**Figure 3:**
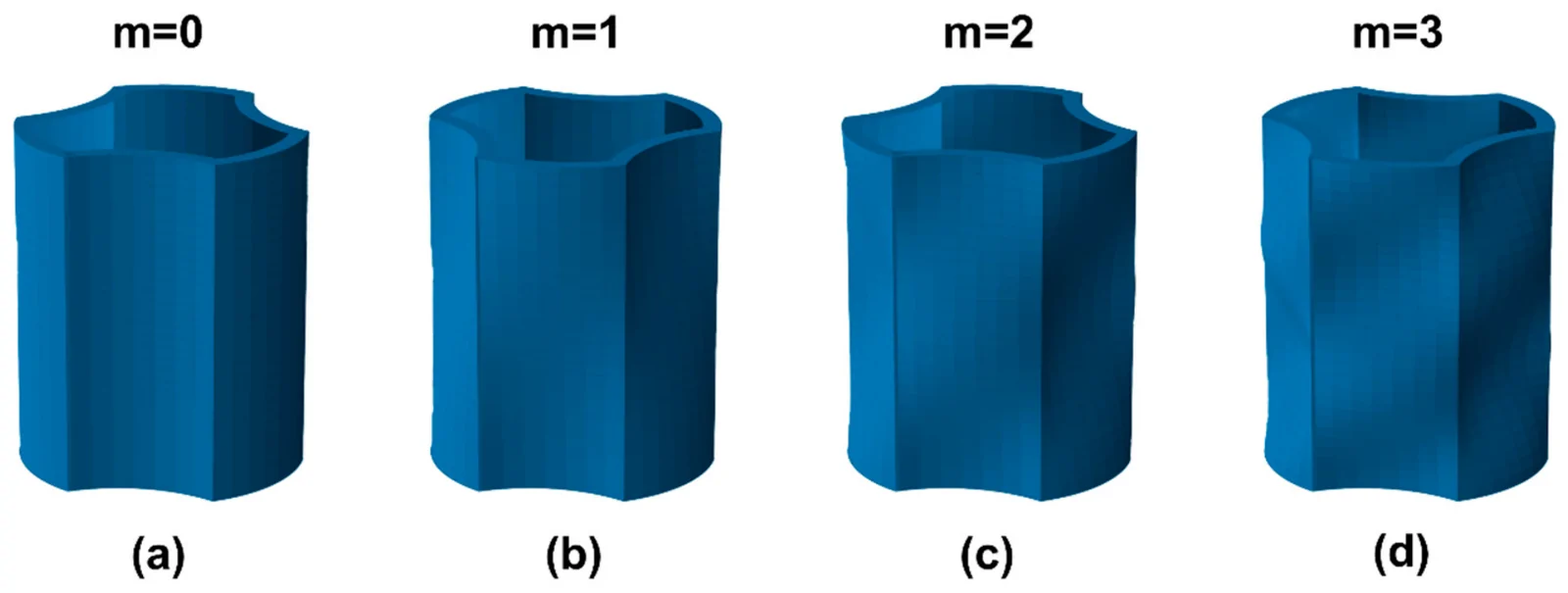
Representative geometries generated by the analytical formulation for (**a**) m=0 (SFS), (**b**) m=1 (SSC), (**c**) m=2, and (**d**) m=3 (SDC).

The same operation is applied locally to the six curved sides, with the modulation direction determined by the corresponding local normal. The resulting formulation preserves the parent two-dimensional topology while introducing controlled three-dimensional variation of the intercellular contact surface. Representative full unit-cell geometries generated in the analytical formulation are shown in Figure 3.

### 2.4. Curvature-Waviness Design Matrix

The CAD modeling and mathematical formulation jointly define a two-parameter design space G(α,m). Keeping m constant while varying α allows us to isolate the effect of the wall curvature within a given family of contact surfaces, while keeping α constant and varying m allows us to compare different contact morphologies along the entire height for the same curvature angle.

**Figure 4:**
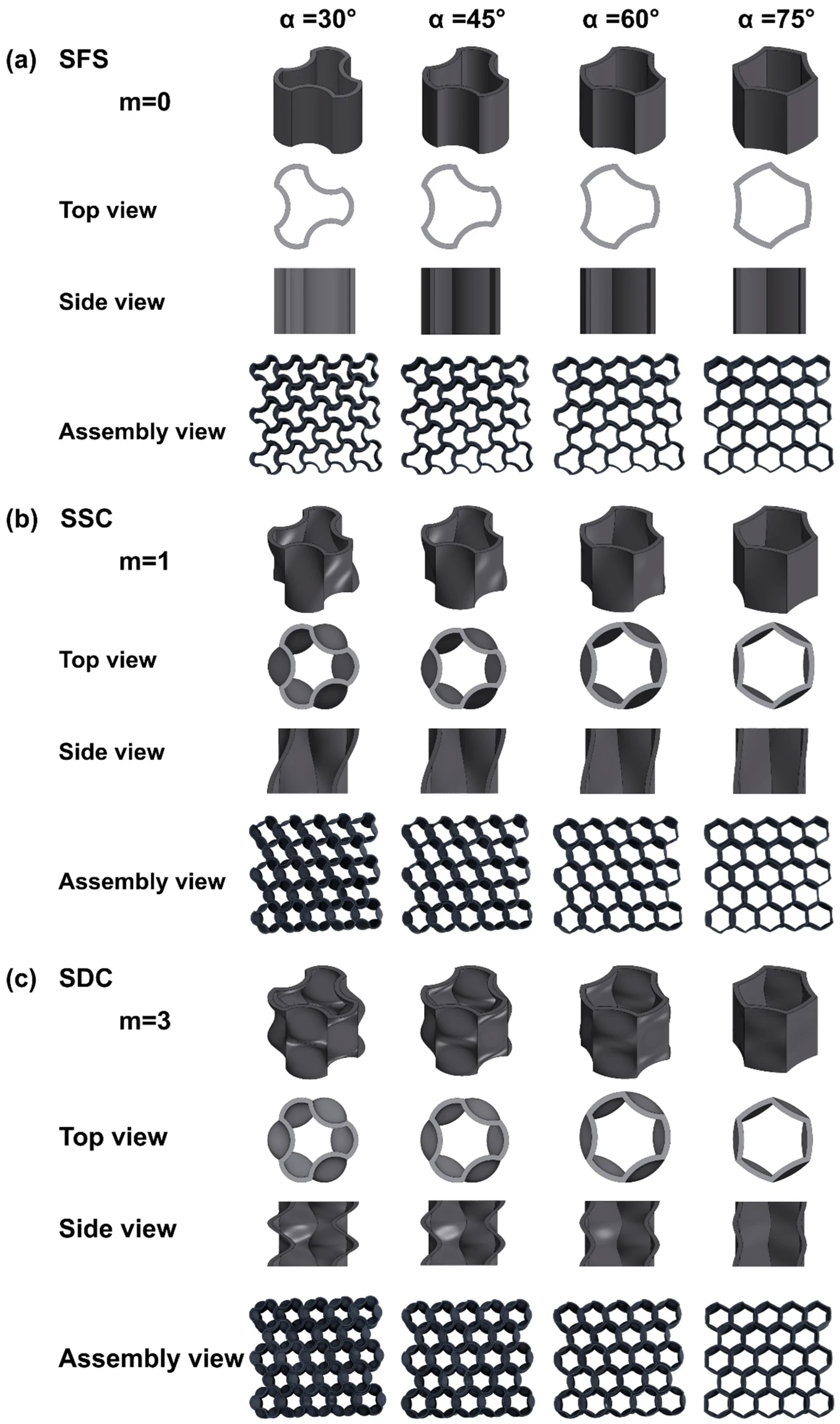
Curvature-waviness design matrix comprising (**a**) SFS (m=0), (**b**) SSC (m=1), and (**c**) SDC (m=3) at α=30°, 45°, 60°, and 75°, shown as unit cells, top views, side views, and assembled arrays.

Three families of contact walls are considered: SFS (m=0), SSC (m=1), and SDC (m=3). Combining these families with α=30°, 45°, 60°, and 75° yields a 3×4 matrix of 12 configurations. Accordingly, this matrix is intended to compare representative flat, lower-waviness, and higher-waviness morphologies across the selected curvature range, rather than to identify a global optimum over all admissible values of α and m. This controlled matrix allows for the systematic examination of the effects of curvature and waviness rather than through case-specific geometries. The resulting unit cells, CAD views, and assembled specimens are summarized in Figure 4.

## 3. Materials and Methods

### 3.1. Material Characterization and Fabrication

All cellular and tensile specimens were fabricated from polyamide 12 (PA12) using Multi Jet Fusion (MJF) under the same processing conditions. The MJF process employed a nominal layer thickness of 0.2 mm and an average printing time of approximately 2–3 s per layer, infill density and infill pattern were not applicable, and no post-processing treatment was applied. The tensile and cellular specimens were fabricated using the same build orientation. Experimental characterization of MJF-processed PA12 is important because its mechanical properties can differ from nominal bulk-polymer values and may depend on the manufacturing conditions [43,44,45].

The PA12 material properties adopted in the present study were obtained from the tensile characterization reported in our previous work [42]. Three dog-bone specimens, manufactured under the same conditions as the cellular specimens, were prepared according to ISO 527-4:2021 [46] and tested under uniaxial tension using an MTS universal testing machine (MTS Systems Corporation, Eden Prairie, MN, USA). The individual stress-strain responses reported in Ref. [42] showed similar mechanical behavior. The corresponding average properties were therefore retained for the present FE simulations: density of 1285 kg/m^3^, Young’s modulus of 979.88 MPa, Poisson’s ratio of 0.35, and yield stress of 14.85 MPa.

The compression specimens were assembled as arrays of discrete self-locking unit cells. The design matrix comprised the SFS (m=0), SSC (m=1), and SDC (m=3) contact-wall families at curvature angles of 30°, 45°, 60°, and 75°. A commercial nylon-specific rapid-curing adhesive, JL-466 (Juli (Dongguan) New Material Technology Co., Ltd., Dongguan, China) was applied locally during assembly to maintain the prescribed initial positions of neighboring cells. Approximately one drop was dispensed using the supplied capillary applicator at each initially contacting cell-to-cell face. The neighboring cells were then brought into their prescribed relative positions and held briefly to establish the initial joint. After assembly, the specimens were left undisturbed for approximately 30 min before compression testing. The adhesive was applied locally at the initial contact regions and served primarily as an assembly constraint rather than as a continuous structural bond over the complete cell surfaces. The cured adhesive-layer thickness and actual local bonded area were not independently measured and therefore cannot be quantified retrospectively.

### 3.2. Quasi-Static Compression Tests

**Figure 5:**
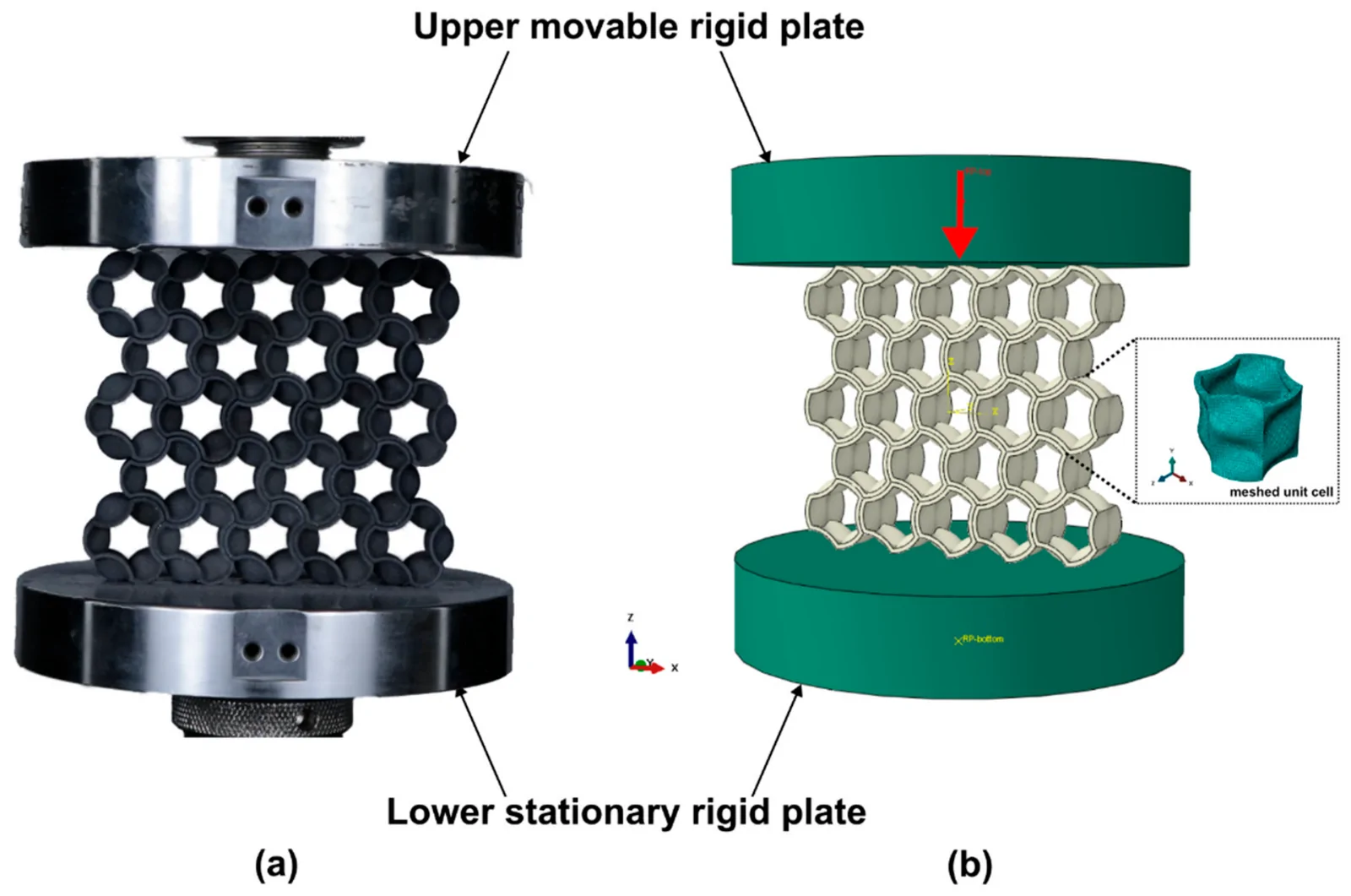
Experimental and numerical compression configurations: (**a**) frameless quasi-static compression specimen positioned between the upper movable and low stationary platens; (**b**) corresponding FE assembly and representative mesh of an individual unit cell.

Quasi-static in-plane compression tests were conducted on the assemblies using a universal testing machine. The specimens were compressed directly between parallel rigid platens without lateral frame confinement, as shown in Figure 5a, thereby allowing intercellular sliding, local rotation, recontact, row misalignment, and progressive compaction to develop freely during loading. The lower platen remained stationary, while the upper platen was displaced vertically at a crosshead speed of 5 mm/min. A front view camera and external lighting system were used to record the deformation sequence throughout each test. These recordings were subsequently used to distinguish progressive compaction from interrupted deformation based on visible features such as progressive void closure, row misalignment, diagonal deformation, and non-uniform compaction. Local sliding, rotation, separation, and recontact were interpreted with support from the FE simulations. Testing was continued into the late compression stage, including the onset of the steep force increase associated with compaction when this stage was reached.

Three independent repetitions of the complete 12-configuration matrix were initially fabricated and tested. The final dataset therefore contains three valid repetitions for each configuration. The reported mechanical quantities are presented as the mean of the three measurements, with variability expressed as the sample standard deviation.

### 3.3. Data Reduction and Performance Metrics

The initial portion of each force-displacement record contained seating effects associated with platen contact and removal of assembly slack. Accordingly, the initial seating region was excluded, and the force-displacement response was re-referenced to the beginning of the compression response.

The structural response was evaluated directly from the measured force-displacement record F(δ). The engineering strain was defined as

(15) $$\varepsilon_{i}=\frac{\delta_{i}}{L}$$

where $\delta_{i}$ is the crosshead displacement after seating correction and L is the initial specimen dimension in the loading direction.

For the early-stage peak response comparisons, the peak force $F_{peak}$ was defined as the maximum force reached within 0≤ε≤15%. To account for differences in specimen mass, the mass-normalized peak force was calculated as

(16) $$F_{peak,n}=\frac{F_{peak}}{m_{s}}$$

where $m_{s}$ is the specimen mass.

EA was evaluated from the area under the force-displacement curve. For a prescribed strain $\varepsilon^{*}$, the absorbed energy was calculated as

(17) $$EA\left(\varepsilon^{*}\right)=\int_{0}^{\delta^{*}}F\left(\delta \right)\;d\delta ,\;\delta^{*}=\varepsilon^{*}L$$

and numerically evaluated from the discrete force-displacement data using the trapezoidal rule. The specific EA was obtained by normalizing the absorbed energy by the specimen mass $m_{S}$:

(18) $$SEA(\varepsilon^{*})=\frac{EA(\varepsilon^{*})}{m_{S}}$$

The reported comparisons focus on $\varepsilon^{*}=15\%$ to characterize the early crushing stage and $\varepsilon^{*}=45\%$ to provide a common late-stage comparison before or during compaction, depending on the deformation mode.

The initial structural stiffness $k_{0}$ was determined from the slope of the force-displacement curve over a fixed strain interval ε=0.5%,3%. A linear regression was applied using the same fitting interval for all specimens without case-specific adjustment. The corresponding mass-normalized initial stiffness was defined as

(19) $$\frac{k_{0}}{m_{S}}={\left.\frac{1}{m_{S}}\;\frac{dF}{d\delta }\right|}_{\varepsilon \in \left[0.5\%,3\%\right]}$$

To quantify crushing-force stability at a prescribed strain, the mean crush force (MCFE) up to that strain was calculated as

(20) $$\bar{F}(\varepsilon^{*})=\frac{1}{\delta^{*}}\int_{0}^{\delta^{*}}F\left(\delta \right)\;d\delta =\frac{EA(\varepsilon^{*})}{\delta^{*}}$$

where $\delta^{*}$ is the displacement corresponding to the prescribed engineering strain $\varepsilon^{*}$. The CFE was defined as

(21) $$CFE(\varepsilon^{*})=\frac{\bar{F}(\varepsilon^{*})}{F_{max}(\varepsilon^{*})}$$

where $F_{max}(\varepsilon^{*})$ is the maximum measured force up to the prescribed strain. SEA and CFE were evaluated at the same prescribed strain levels used for the reported comparisons. This permits direct comparison at 15% and 45% strain without treating 45% strain as a universal densification strain.

### 3.4. Finite-Element Model

FE simulations were performed in ABAQUS/Explicit 2022 to support interpretation of the experimentally observed load-transfer and compaction mechanisms. A 5×5 assembly matching the experimental geometry was positioned between rigid upper and lower platens. The lower platen was fully constrained, and the upper platen was prescribed a vertical displacement while its lateral translations and rotations were restricted.

The printed PA12 cells were modeled as a homogeneous isotropic elastoplastic material using the experimentally characterized response described in Section 3.1 and Ref. [42]. This representation does not explicitly account for MJF-induced anisotropy, porosity, local surface heterogeneity, viscoelastic or viscoplastic effects, strain-rate dependence, or thermal effects. Moreover, local strains in curved walls, contact regions, and plastic hinges can substantially exceed the global assembly strain during compaction. The FE model was therefore used primarily to reproduce the global load-transfer and contact-evolution mechanisms rather than the complete local material response under severe deformation.

Normal interaction between contacting cell surfaces was described using hard contact, while tangential interaction was represented using a Coulomb friction coefficient of μ=0.30. The same nominal coefficient was previously employed for the corresponding MJF-processed PA12 self-locking system in Ref. [42]. Tribological studies of additively manufactured PA12 have shown that the friction coefficient depends on surface condition, build orientation, normal load, and sliding conditions [47,48]. More specifically, Gavcar et al. reported coefficients of friction up to approximately 0.355 for MJF-processed PA12 under dry ball-on-disc conditions [49]. Given the differences between those tribological test conditions and the evolving PA12-to-PA12 contacts within the present cellular assembly, μ=0.30 is treated here as an effective numerical contact parameter rather than as a directly measured property of the present specimens.

The adhesive-assisted initial constraint between neighboring cells was represented by effective cohesive interactions assigned to the initially joined interfaces. The cohesive formulation permits progressive loss of the initial constraint followed by frictional sliding, separation, and recontact. Since the cured JUL/JL-466 adhesive-layer thickness, actual bonded area, and interface constitutive response were not independently characterized, the formulation was not intended as a calibrated material model of the adhesive, but as an effective representation of the initial assembly constraint. The adopted parameter set, previously employed in Ref. [42], is summarized in Table 2.

The cellular geometry was discretized using 10-node modified quadratic tetrahedral elements (C3D10M) with a nominal element size of 1 mm. Explicit time integration was used to accommodate large deformation, local instability, separation, sliding, and evolving contact. The kinetic to internal energy ratio remained below 5% throughout the analyses, providing a quasi-static check. The FE assembly and representative mesh are shown in Figure 5b. The simulations were used to interpret local contact retention, sliding, separation, and recontact, whereas row misalignment and progressive compaction were identified directly from the experimental deformation sequences.

Mesh convergence was assessed using the SDC 45° configuration as a representative case because it combines the more complex SDC contact morphology with progressive compaction. Nominal C3D10M element sizes of 1.50, 1.00, and 0.70 mm were examined under identical material, contact, boundary, and loading conditions. Convergence was evaluated from the force-displacement response, initial structural stiffness, early-stage peak force, and SEA at 45% strain. The percentage change between successive mesh levels was calculated relative to the finer discretization, and the results are summarized in Table 3.

Refinement from 1.50 to 1.00 mm resulted in changes of 0.57%, 7.72%, and 8.68% in $k_{0}$, $F_{peak}$ and SEA, respectively. Further refinement to 0.70 mm reduced the corresponding differences to 3.68%, 0.24%, and 1.51%. The 1.00 mm mesh was therefore retained because the principal global response quantities were sufficiently stable, and the 0.70 mm discretization could not be generated consistently for all twelve geometries owing to geometry-dependent meshing limitations.

To assess the sensitivity of the numerical response to the assumed friction coefficient, additional simulations were performed for the representative SDC 45° configuration using μ=0.20, 0.30, and 0.40. The nominal value μ=0.30 was treated as the reference, while all material properties, cohesive interface parameters, mesh, boundary conditions, and loading conditions were kept unchanged. Sensitivity was evaluated using $F_{peak}$, SEA at 45% strain, and the force at 45% strain, $F_{45\%}$. The results are summarized in Table 4.

Within the investigated range, $F_{peak}$ and SEA at 45% strain showed comparatively small variations, whereas $F_{45\%}$ exhibited a stronger dependence on friction. This indicates that the later-stage force response is more sensitive to intercellular friction as contact engagement and compaction develop. Nevertheless, the characteristic late-stage force increase was retained for all three coefficients. Thus, μ=0.30 provides a suitable nominal value for the present mechanism-oriented FE interpretation, while the absolute force level during later-stage compaction remains friction dependent.

## 4. Results and Discussion

The compression response is discussed in terms of the two prescribed geometrical variables introduced in Section 2: the in-plane curvature angle α and the through-height waviness index m. The force-displacement curves are first examined by contact wall family and by curvature angle, followed by the early response indicators SEA and CFE at common strain levels. Finally, the densification observations, geometrical engagement descriptors, and finite element results are combined to interpret how increasing contact engagement changes the stability of progressive compaction.

### 4.1. Force-Displacement Response

**Figure 6:**
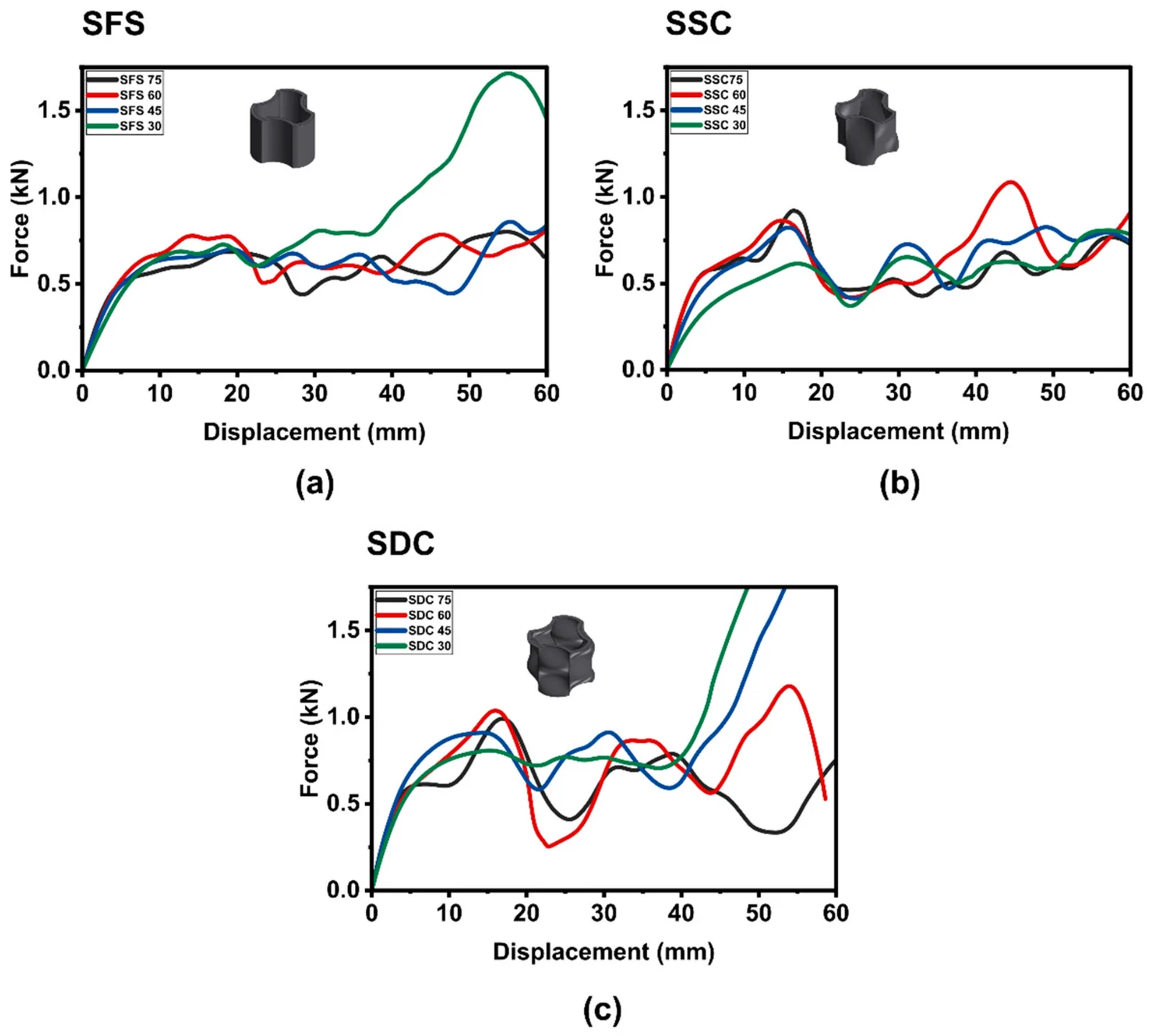
Experimental force-displacement responses grouped by contact wall family: (**a**) SFS (m=0), (**b**) SSC (m=1), and (**c**) SDC (m=3).

Figure 6 compares the force-displacement responses within each contact wall family. All configurations exhibit an initial nonlinear rise followed by a fluctuating crushing regime, but the force level and late-stage evolution depend strongly on both α and m. Geometry-dependent transitions in load-bearing response and failure mode have likewise been reported for honeycomb structures under in-plane compression [50], while reinforcement of the load-bearing wall geometry can substantially alter mechanical response and EA [51].

SFS (m=0) is the most compliant family. Its flat through-height contact morphology allows greater relative sliding and rotation between neighboring cells, reducing early load transfer. Mean early-stage mass-normalized peak force ranges from 8.97 to 12.54 N/g, with SFS 75° giving the lowest value.

Adding through-height waviness increases resistance to relative motion. SSC (m=1) therefore occupies a transitional force range between SFS and SDC, although its relative ranking depends on curvature. Its early response strengthens as α increases from 30° to 60°, but the 75° configuration does not continue this increase. Curvature and engagement consequently do not produce a monotonic gain in load capacity.

SDC (m=3) reaches the highest force levels overall. Mean $F_{peak}/m_{s}$ of 16.71 and 17.11 N/g are obtained for SDC 45° and SDC 60°, respectively, the latter being the maximum in the design matrix. Post-peak oscillations are also more pronounced than in the SFS family. The FE simulations indicate that these fluctuations are associated with repeated engagement, release, sliding, and recontact between adjacent cells rather than with wall bending alone.

**Figure 7:**
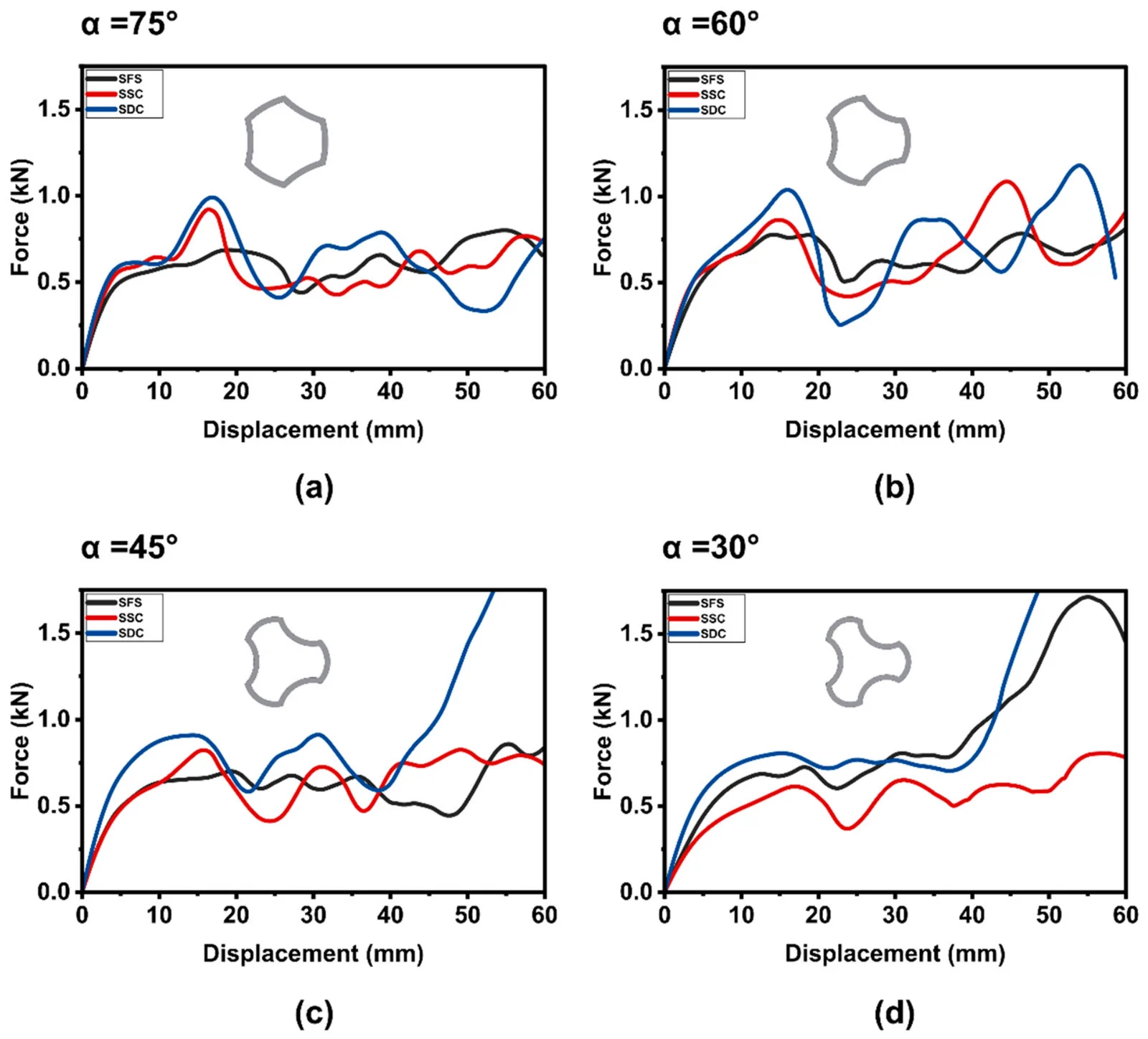
Experimental force-displacement responses grouped by curvature angle: (**a**) 75°, (**b**) 60°, (**c**) 45°, and (**d**) 30°.

Regrouping the same data by curvature angle (Figure 7) isolates the effect of waviness at fixed α. At 30°, SDC sustains the highest force over much of the early and intermediate response; the mean early-stage mass-normalized peak forces are 14.07, 12.19, and 10.51 N/g for SDC, SFS, and SSC, respectively. At 45°, the separation becomes clearer. SDC 45° reaches a mean $F_{peak}/m_{s}$ of 16.71 N/g while maintaining a comparatively high crushing force through the early and intermediate stages.

At 60°, each family shows a different advantage: SDC gives the highest peak resistance, SSC develops high initial stiffness, and SFS retains a smoother post-peak trajectory. Raising α to 75° does not improve any family relative to its best intermediate-angle case. SFS 75° in particular records the lowest mass-normalized peak force in the matrix. The influence of α is therefore non-monotonic.

### 4.2. Initial Stiffness and Peak Response

**Figure 8:**
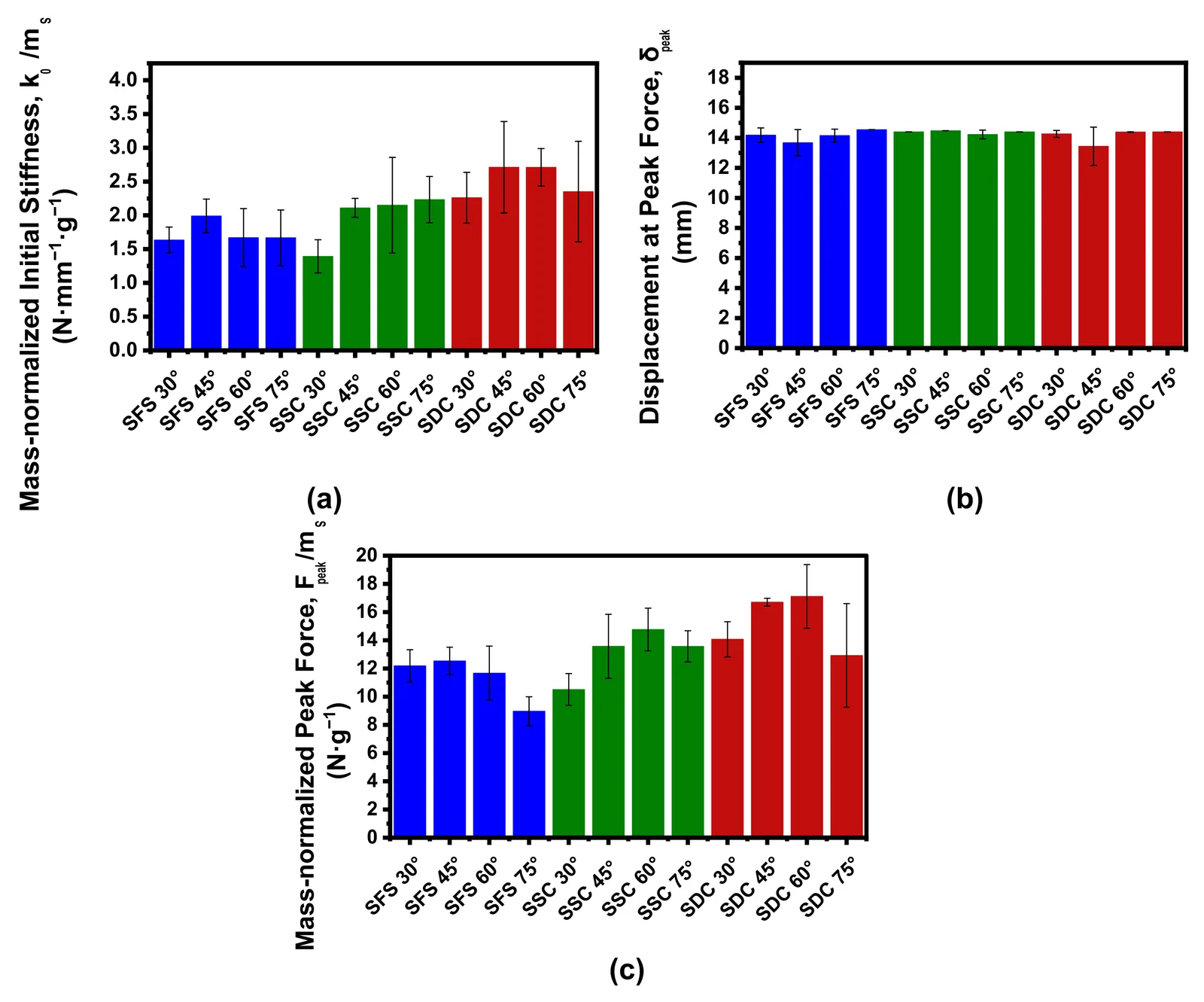
Early-stage mechanical indicators of the twelve configurations: (**a**) mass-normalized initial stiffness, (**b**) displacement at peak force, and (**c**) mass-normalized peak force. Error bars show the sample standard deviation of the three repeated tests.

Figure 8 summarizes the early-stage mechanical indicators. The SFS family exhibits mean mass-normalized initial stiffness ($k_{0}/m_{s}$) values ranging from 1.63 to 1.99 N·mm^−1^·g^−1^. For the SSC family, $k_{0}/m_{s}$ increases from 1.39 N·mm^−1^·g^−1^ at 30° to 2.11 N·mm^−1^·g^−1^ at 45°, followed by smaller increases to 2.15 and 2.23 N·mm^−1^·g^−1^ at 60° and 75°, respectively.

The SDC family remains at a higher stiffness level, with mean $k_{0}/m_{s}$ values ranging from 2.26 to 2.71 N·mm^−1^·g^−1^. The highest mean $k_{0}/m_{s}$ values are obtained for SDC 45° and SDC 60°, both reaching $2.71\;N\cdot {mm}^{-1}\cdot g^{-1}$, while SDC 60° reaches the highest mean $F_{peak}/m_{s}$ of 17.11 N/g.

Family averages further indicate the influence of through-height waviness. The mean $k_{0}/m_{s}$ increases from 1.74 N·mm^−1^·g^−1^ for SFS to 1.97 N·mm^−1^·g^−1^ for SSC and 2.51 N·mm^−1^·g^−1^ for SDC. The effect of curvature remains non-monotonic. Averaged across the three families, $k_{0}/m_{s}$ rises from 1.76 N·mm^−1^·g^−1^ at 30° to 2.27 N·mm^−1^·g^−1^ at 45°, and then decreases to 2.18 and 2.08 N·mm^−1^·g^−1^ at 60° and 75°, respectively. The mass-normalized peak force follows the same broad pattern, increasing from 12.26 to 14.52 N/g before falling to 11.82 N/g. Through-height waviness therefore provides a clearer increase in early resistance, whereas curvature produces a limited range benefit rather than a continuous increase.

### 4.3. Specific Energy Absorption and Crushing Stability

**Figure 9:**
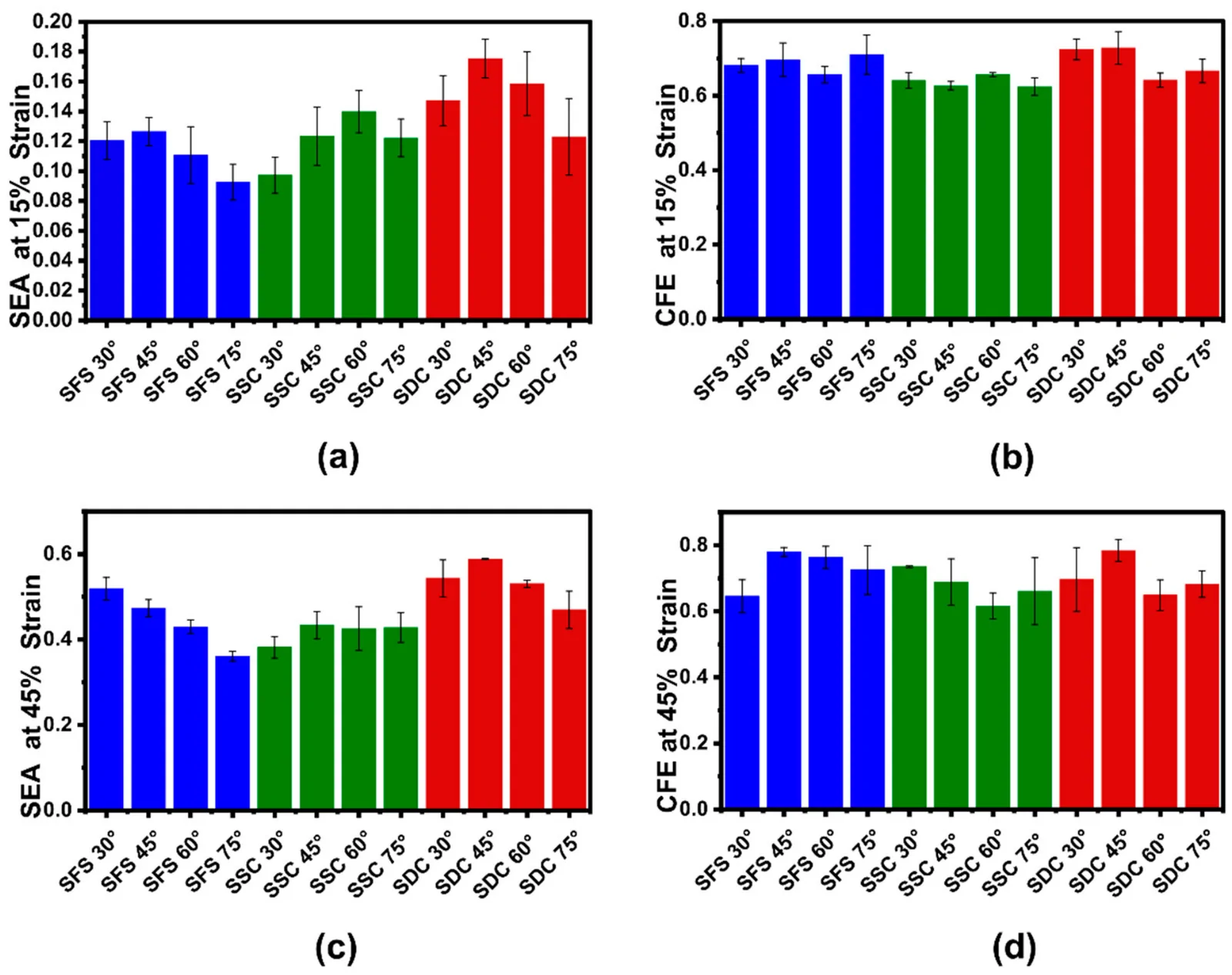
Specific energy absorption and crushing-force efficiency of the twelve configurations at common strain levels: (**a**) SEA at 15% strain, (**b**) CFE at 15% strain, (**c**) SEA at 45% strain, and (**d**) CFE at 45% strain. Error bars show the sample standard deviation of the three repeated tests.

SEA and CFE were evaluated together because high energy absorption does not necessarily imply stable crushing. Figure 9a,b compares the two metrics at 15% strain, which provides a common early-stage deformation level for all specimens. The SDC family provides the highest early SEA, with a family average of 0.15 J/g compared with 0.12 J/g for SSC and 0.11 J/g for SFS. SDC 45° gives the maximum SEA at this strain (0.18 J/g), followed by SDC 60° (0.16 J/g) and SDC 30° (0.15 J/g).

The mean CFE values add an important distinction. SDC 45° also records the highest early-stage CFE (0.73), closely followed by SDC 30° (0.72), showing that its performance is sustained rather than dominated by an isolated peak. SSC, despite its intermediate stiffness and SEA, has the lowest family-average CFE (0.64). SFS 30° and SFS 45° retain relatively high CFE values of 0.68 and 0.69, although their SEA remains lower.

Figure 9c,d gives the late-stage comparison at 45% strain, used here as a common evaluation point rather than a universal densification strain. The configurations reach densification at different stages; therefore, the SEA and CFE values evaluated at 45% strain do not necessarily represent the same physical compaction state for all designs. At this strain, some configurations are approaching or undergoing densification, while others remain in progressive crushing or exhibit interrupted compaction. Accordingly, the 45% values are used as common fixed deformation measures for comparing energy absorption and crushing-force efficiency, whereas the configuration of specific densification behavior is assessed separately in Section 4.4. SDC 45° gives the highest mean SEA within the investigated matrix at 0.59 J/g, followed by SDC 30° (0.54 J/g) and SDC 60° (0.53 J/g). Family averages are 0.53 J/g for SDC, 0.44 J/g for SFS, and 0.42 J/g for SSC. SFS 75° gives the lowest mean value, 0.36 J/g, consistent with its low force level and interrupted late-stage compaction.

For CFE at 45% strain, SDC 45° gives the highest mean value (0.78), closely followed by SFS 45° (0.77) and SFS 60° (0.76). At the family level, however, SFS gives the highest MCFE (0.73), followed by SDC (0.70) and SSC (0.67). This distinction shows that the geometry providing the highest energy absorption does not necessarily correspond to the family with the highest overall crushing-force efficiency.

Since only three repetitions were available for each configuration, the comparisons in Figure 8 and Figure 9 are interpreted primarily in terms of mean response and experimental scatter. Overlap between the reported standard deviations indicates that small differences between individual configurations should not be interpreted as statistically significant rankings. Accordingly, the discussion emphasizes broader trends associated with curvature and waviness, while statements concerning individual configurations refer to the highest or lowest mean values within the investigated matrix.

### 4.4. Densification and Deformation Modes

The fixed strain metrics do not fully describe whether a specimen develops progressive compaction. To provide a more reproducible classification of the late-stage response, each experimental repetition was evaluated separately. A densification candidate was identified from the maximum of the energy absorption efficiency curve within the late-stage strain range of 30–75%. The candidate was accepted only when it occurred before the end of the recorded response and was followed by a sustained load increase of at least 2% over the subsequent 3% strain interval. The recorded deformation sequence was then used to confirm that the late-stage response corresponded to continued compaction rather than loss of contact or non-uniform deformation. When the deformation sequence did not provide sufficiently clear evidence of densification, the repetition was conservatively classified as not reached.

Based on the three repeated tests, a configuration was classified as progressive densification when all three repetitions developed sustained coherent compaction, as case-dependent densification when this behavior occurred in only one or two repetitions, and as interrupted compaction when all three repetitions exhibited disruption of the compaction process before coherent densification, including cell loss/ejection, row misalignment, or loss of the intercellular load path. The repetition level outcomes and resulting classifications are summarized in Table 5.

**Figure 10:**
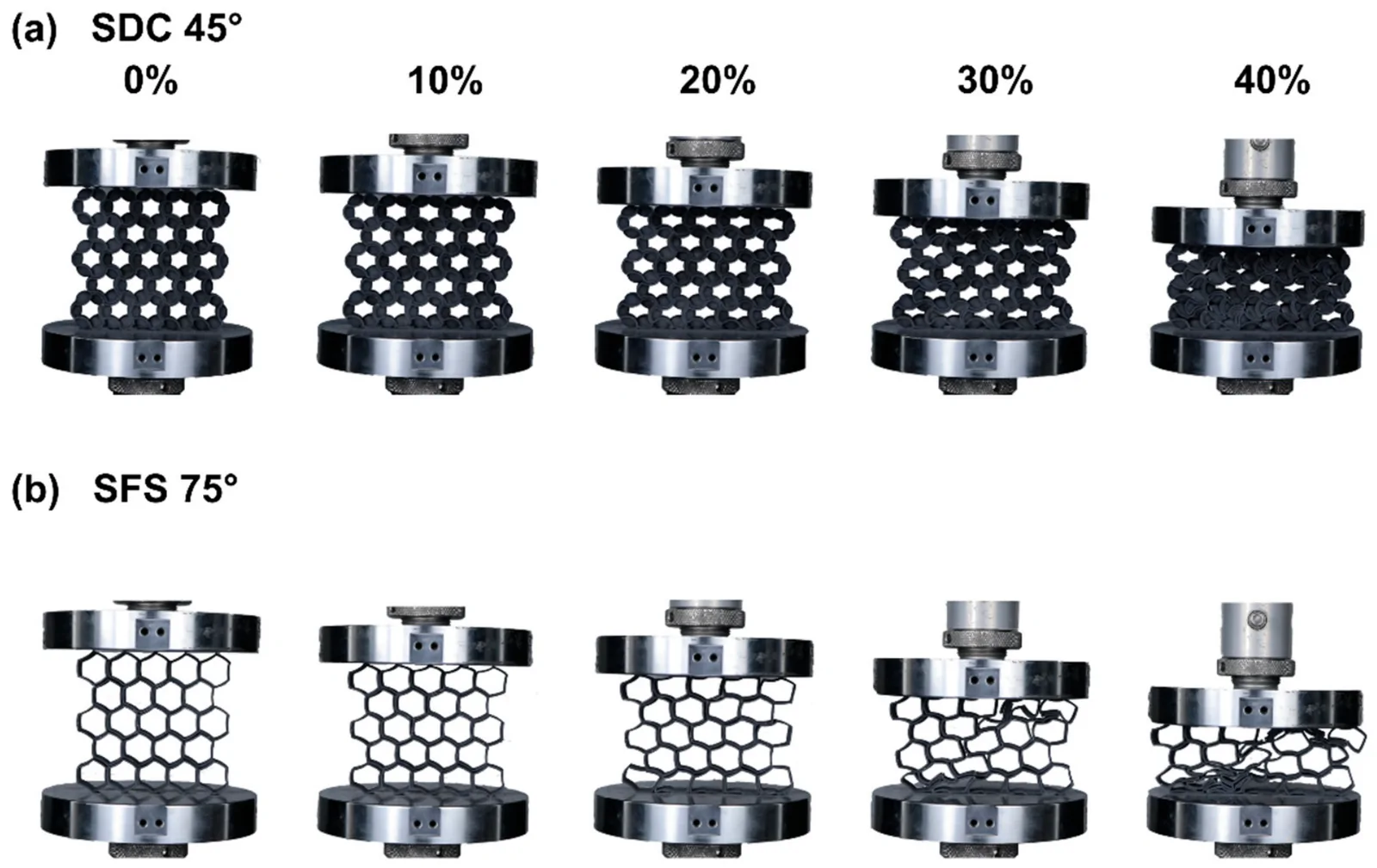
Representative experimental deformation sequences at 0%, 10%, 20%, 30%, and 40% engineering strain: (**a**) SDC 45°, showing progressive coherent compaction, and (**b**) SFS 75°, showing diagonal deformation, row misalignment, and interrupted compaction.

The deformation sequences of SDC 45° and SFS 75° provide representative examples of the two limiting behaviors, shown in Figure 10. SDC 45° remains laterally coherent during progressive compression and gradually closes its internal void space, with most rows remaining engaged as the specimen approaches a compacted state. SFS 75° evolves differently: local distortion is followed by a diagonal, shear-like deformation path, row misalignment, and non-uniform compaction, leaving some regions highly compressed while others remain open or tilted.

These observations support a contact path interpretation of the late-stage response. Stable compaction is associated with sufficient intercellular engagement to transfer load while retaining enough local freedom for sliding, rotation, recontact, and void closure. When relative motion develops into row misalignment or partial loss of the intercellular contact network, the imposed displacement can increase without producing uniform densification.

### 4.5. Geometrical Engagement Descriptors

To relate the prescribed geometry to the observed compaction behavior, two dimensionless geometric descriptors are considered. The contact area index normalizes the one-sided contact wall surface area $A_{C}(\alpha ,m)$ by the projected flat reference area aH:

(22) $$\Lambda_{A}=\frac{A_{C}(\alpha ,m)}{\left(aH\right)}$$

**Figure 11:**
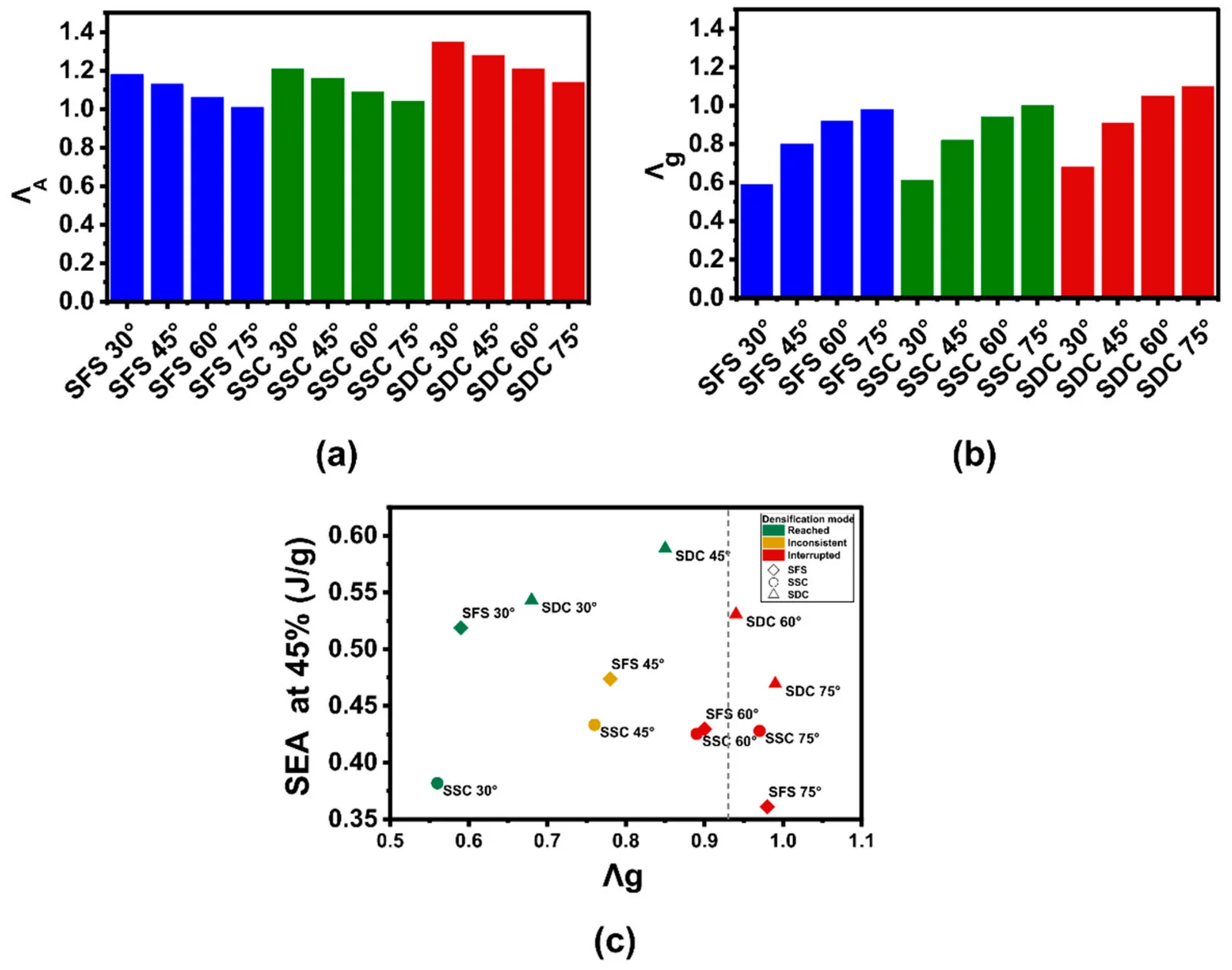
Geometrical engagement descriptors and late-stage performance: (**a**) contact area index $\Lambda_{A}$, (**b**) angularly weighted engagement descriptor $\Lambda_{g}$, and (**c**) mean SEA at 45% strain from three repeated tests as a function of $\Lambda_{g}$, with symbols and colors indicating contact wall family and observed densification mode.

In the present dataset, $A_{C}(\alpha ,m)$ was obtained from the corresponding 3D parametric contact surface defined in Section 2.3 and reported in Figure 2. Because contact area alone does not account for the orientation of the curved wall relative to the loading direction, an angularly weighted engagement descriptor is defined as

(23) $$\Lambda_{g}=\Lambda_{A}\sin\alpha$$

where α is the curvature angle. The factor sinα introduces a first-order orientation weighting to account for the fact that intercellular load transfer depends not only on the available contact area but also on the orientation imposed by the wall curvature relative to the loading direction. In this sense, sinα provides a simplified measure of the directional contribution of contact engagement. Because the local surface orientation varies over the curved contact surface, this weighting does not represent an exact local force projection. It is not an additional area contribution. The two descriptors are examined in Figure 11.

Angular weighting produces a clearer ordering of the design space. The low and intermediate engagement region contains the configurations that reached densification, whereas most interrupted cases occupy the higher engagement region. The relationship between $\Lambda_{g}$ and SEA at 45% strain is non-monotonic: the largest late-stage SEA occurs for SDC 45° (0.59 J/g), while configurations with still stronger engagement do not provide a corresponding increase in SEA and are predominantly classified as interrupted.

This trend is consistent with the deformation observations and suggests that insufficient engagement can limit load transfer, whereas excessive engagement can restrict the local sliding and rotation needed for progressive void closure and instead promote localized release, misalignment, or non-uniform compaction. $\Lambda_{g}$ should therefore be treated as a geometry-based organizing descriptor rather than as a universal stability criterion. Its apparent transition region is specific to the present PA12 material, wall thickness, interface condition, fabrication route, and frameless quasi-static loading condition.

To further quantify the relationship between the geometric descriptors and the mechanical response, Spearman correlation coefficients were calculated across the twelve configurations for mass-normalized initial stiffness, mass-normalized $F_{peak}$, SEA at 45% strain, and CFE at 45% strain. The coefficient ranges from −1 to 1, where positive and negative values indicate increasing and decreasing monotonic trends, respectively, while values close to zero indicate a weak monotonic relationship. The results are summarized in Table 6.

Among the evaluated response quantities, $\Lambda_{A}$ shows the strongest positive association with SEA at 45% strain, with a correlation coefficient of 0.704. The remaining correlations are weaker, although $\Lambda_{g}$ shows a moderate positive association with initial stiffness. These results indicate that the descriptors capture selected trends in the mechanical response but do not provide a simple monotonic prediction of all performance metrics. This is consistent with the non-monotonic engagement compaction behavior observed across the investigated configurations.

### 4.6. FE-Supported Mechanism Interpretation

**Figure 12:**
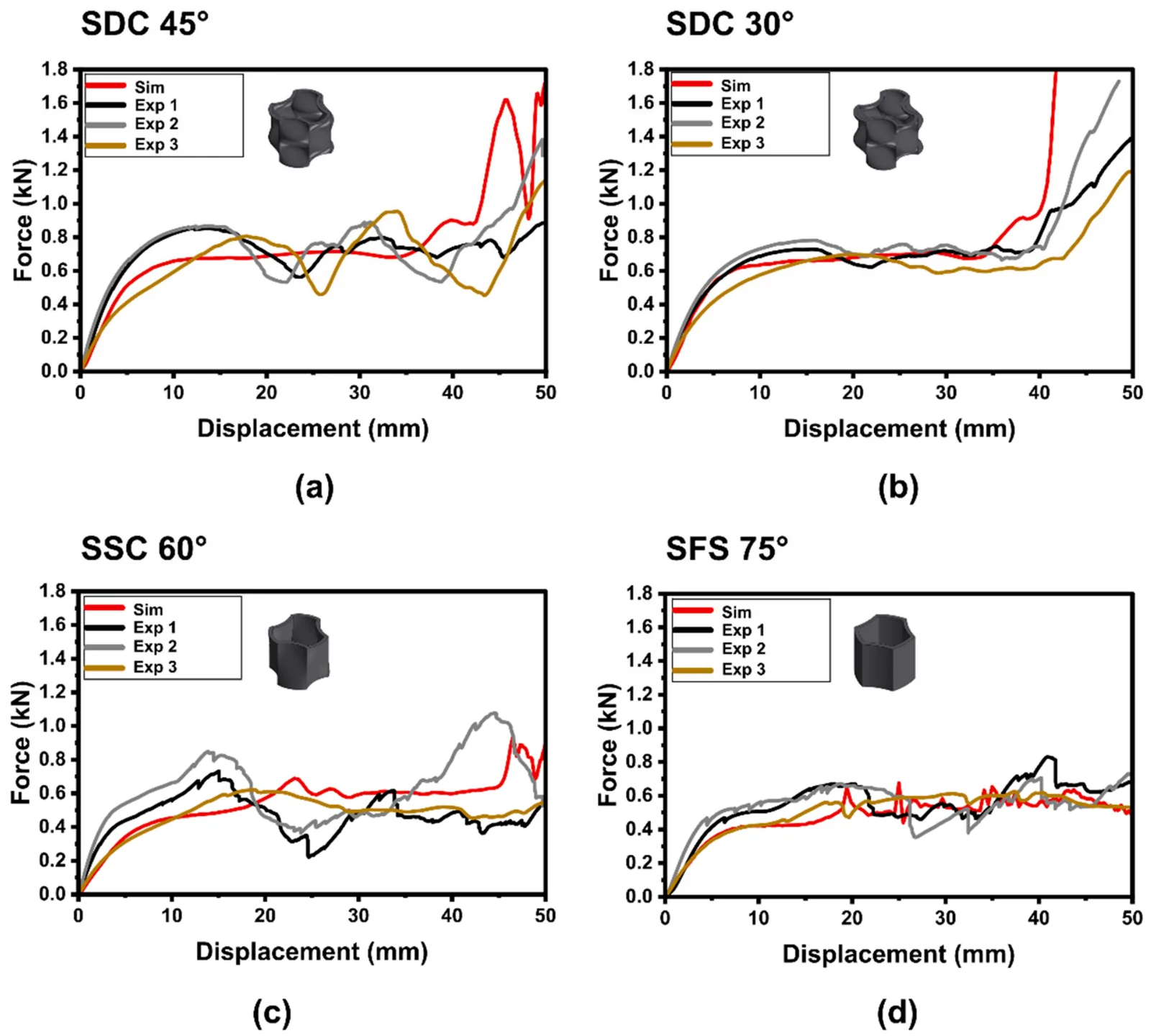
Experimental and finite element force-displacement comparisons for representative configurations: (**a**) SDC 45°, (**b**) SDC 30°, (**c**) SSC 60°, and (**d**) SFS 75°.

FE simulations were used to determine whether the idealized model reproduces the principal response tendencies associated with the observed compaction modes. Figure 12 compares the numerical and experimental force-displacement curves for four representative configurations: SDC 45° and SDC 30° as contact retaining cases, SSC 60° as an intermediate fluctuating case, and SFS 75° as an interrupted compaction case.

For SDC 45°, the simulation reproduces the rapid initial rise and the experimental force range over much of the early and intermediate deformation interval. The numerical late-stage force increases more sharply than in the experiments, indicating an overprediction of final locking or densification stiffness. SDC 30° shows a similar trend: both experiment and simulation exhibit a stable plateau followed by a late force increase, although the numerical densification rise occurs more abruptly. Part of the late-stage discrepancy can be associated with the constitutive idealization of the printed PA12. The homogeneous isotropic elastoplastic model is calibrated from uniaxial tensile characterization and does not reproduce the full process-dependent and time-dependent response of MJF PA12 under repeated local bending, plastic-hinge formation, frictional contact, and severe local strain accumulation. During densification, these localized deformation mechanisms can produce progressive redistribution and relaxation that are not fully represented by the present constitutive description. The resulting numerical response can therefore become artificially stiff once extensive wall-to-wall contact and geometric locking develop. This provides a plausible material-level contribution to the sharper numerical densification rise observed in Figure 12, in addition to the idealized geometry and interface assumptions.

SSC 60° is reproduced at the level of the overall force range, but the model does not capture the specimen-specific sequence of local drops and recoveries. That difference is consistent with repeated sliding and recontact indicated by the FE simulations and with the corresponding load fluctuations observed experimentally. For SFS 75°, the simulation stays in the lower experimental force range and, unlike the densifying SDC cases, does not develop a pronounced late-stage rise.

To quantify the agreement shown in Figure 12, Table 7 compares the initial structural stiffness, early-stage peak force, SEA at 45% strain, and the displacement corresponding to the onset of the late-stage force increase for the four representative configurations. Experimental values are reported as the mean ± sample standard deviation of the three repetitions. For SSC 60° and SFS 75°, no sustained late-stage force rise was identified because progressive compaction was interrupted; consequently, no onset displacement or percentage difference was assigned for this quantity.

The quantitative comparison shows that the level of agreement depends on both configuration and response metric. For SDC 45° and SDC 30°, the differences in initial stiffness, peak force, and the onset displacement of the late-stage force increase remain within approximately 8–18%, while the corresponding SEA differences are 2.1% and 10.5%, respectively. Larger differences in initial stiffness and peak force are observed for SSC 60° and SFS 75°, reaching approximately 24–32%, whereas the SEA values remain more closely reproduced. For these latter configurations, the absence of a sustained late-stage rise is consistent with their interrupted compaction response.

The numerical comparison should therefore be interpreted primarily as mechanism support rather than point-by-point validation. The model uses idealized geometry, a homogeneous isotropic PA12 representation, nominal friction, and effective cohesive interface parameters. It does not explicitly reproduce process-induced anisotropy, porosity, surface roughness, local adhesive distribution, geometric mismatch, viscoelastic/viscoplastic relaxation, or specimen-specific recontact. These simplifications contribute to the smoother numerical response and may explain part of the overprediction of late-stage locking in the strongly compacting configurations. However, the simulations reproduce the principal distinction between sustained contact retaining compaction and interrupted load path development and therefore remain useful for interpreting the geometry-dependent deformation mechanisms.

Overall, the response is non-monotonic. Increasing m generally raises early stiffness and peak resistance by strengthening 3D intercellular engagement, whereas increasing α beyond the intermediate range reduces late-stage stability by restricting compatible rearrangement. SDC 60° maximizes peak resistance, but SDC 45° provides the strongest combined SEA, CFE, and densification response within the tested matrix. Maximum resistance and stable progressive compaction are therefore distinct design targets.

## 5. Design Implications and Limitations

### 5.1. Design Implications

The results show that the compressive response of the self-locking honeycombs is governed by the combined effects of in-plane curvature and through-height contact morphology. Increasing the waviness index generally strengthens early intercellular engagement and raises initial stiffness and peak resistance, whereas the effect of curvature angle is non-monotonic. Within the investigated matrix, SDC 60° provides the highest mean mass-normalized $F_{peak}$ of 17.11 N/g, while SDC 45° provides the highest mean SEA and CFE together with stable progressive compaction.

Maximum geometric constraint is not necessarily desirable. Stable compression requires sufficient contact to maintain load transfer while still allowing the local sliding, rotation, recontact, and void closure required for continued compaction. The descriptors $\Lambda_{A}$ and $\Lambda_{g}$ are therefore useful for organizing the geometry-response trends within the present design space, but they should not be interpreted as universal stability criteria.

This behavior provides the main physical distinction from the previous frame-confined investigation [42]. Under confinement, the deformation path was externally restricted, and the effects of curvature and contact wall morphology could not be separated. In the present frameless two-parameter study, these variables are independently controlled, revealing a non-monotonic engagement compaction relationship. Increasing engagement can strengthen early load transfer and peak resistance, whereas excessive constraint can suppress the relative motions required for compatible void closure and thereby promote row misalignment or interrupted compaction. Thus, maximum peak resistance and stable progressive compaction represent distinct design objectives within the investigated self-locking architecture.

### 5.2. Limitations

The present findings should be interpreted within the scope of the experimental and numerical framework. Although three repetitions per configuration demonstrated repeatability, the sample size limits strong ranking where uncertainty ranges overlap. The PA12 material input was adopted from Ref. [42], while specimen-level numerical data for further statistical assessment and the absolute build orientation relative to the MJF machine axes were unavailable. The adhesive-layer thickness and actual bonded area were not independently measured; consequently, the cohesive parameters represent effective interface properties, and no dedicated cohesive-parameter sensitivity analysis was performed. The intercellular friction coefficient was also not measured directly, although the SDC 45° sensitivity study over μ=0.20−0.40 showed comparatively small variations in early-stage peak force and SEA and greater sensitivity in the later-stage force response. Local sliding, separation, and recontact were interpreted with support from the FE simulations rather than measured directly by full-field techniques. The isotropic FE model further omits MJF-induced anisotropy, porosity, roughness, and other manufacturing imperfections, which may contribute to experimental-numerical differences during severe deformation. Finally, the study was limited to quasi-static compression at 5 mm/min; strain-rate effects, structural inertia, and dynamic contact evolution may alter the response under impact conditions, requiring dynamic validation before extending the conclusions to crash-relevant loading.

## 6. Conclusions

A two-parameter geometric framework was developed to systematically control the in-plane curvature angle α and the through-height waviness index m of arc-curved self-locking honeycombs. The formulation generated twelve configurations within a common geometric family and enabled the effects of curvature and contact morphology to be examined independently through frameless quasi-static compression tests and nonlinear finite element simulations.

Increasing m generally enhanced the initial stiffness and peak resistance by strengthening 3D intercellular engagement, while the effect of α remained non-monotonic. SDC 60° produced the highest mean mass-normalized $F_{peak}$ of 17.11 N/g, whereas SDC 45° achieved the highest late-stage specific energy absorption of 0.59 J/g and CFE of 0.78, together with stable progressive compaction. The experimental observations and simulations indicate that effective self-locking behavior requires a balance between intercellular engagement and deformation compatibility. Excessive constraint can restrict sliding, rotation, recontact, and progressive void closure, leading to row misalignment and interrupted compaction. The results therefore show that curvature and contact wall waviness should be coordinated according to the required balance between load resistance, energy absorption, and compaction stability.

## Figures and Tables

**Table 1:** Radius and sagitta associated with the investigated curvature angles.

Curvature Angle α	$R_{\alpha }$ (mm)	$A_{\alpha }$ (mm)
30°	6.928	3.464
45°	8.485	2.485
60°	12.000	1.608
75°	23.182	0.790

**Table 2:** Effective cohesive interface parameters adopted in the present FE model; the parameter set was previously reported in Ref. [42].

Parameter	Symbol	Value
Normal stiffness	$K_{n}$	57 N/mm^3^
Shear stiffness	$K_{s}$	57 N/mm^3^
Normal strength	$\sigma_{n}^{max}$	5.7 MPa
Shear strength	$\tau_{s}^{max}$	5.7 MPa
Mode I fracture energy	$G_{c}^{n}$	0.028 N/mm
Mode II fracture energy	$G_{c}^{s}$	0.028 N/mm
Viscous regularization	$\mu_{visc}$	1 × 10^−5^ s
Mixed-mode exponent	η	1.2

**Table 3:** Mesh-convergence assessment for the representative SDC 45° configuration using C3D10M elements.

Nominal Mesh Size (mm)	$k_{0}$ (N/mm)	$F_{peak}$ (N)	SEA at 45% Strain (J/g)	${\Delta k}_{0}$ (%) ^1^	$\Delta F_{peak}$ (%) ^1^	$\Delta {SEA}_{45\%}$ (%) ^1^
1.50	123.08	726.45	0.606	0.57	7.72	8.68
1.00	122.38	674.38	0.557	3.68	0.24	1.51
0.70	118.04	676.03	0.549	−	−	−

^1^ Percentage change relative to the next finer mesh, $\Delta X=(|X_{coarse}-X_{fine}|/X_{fine})\times 100$.

**Table 4:** Friction coefficient sensitivity of the representative SDC 45° configuration.

μ	$F_{peak}$ (N)	$\Delta F_{peak}$ (%) ^1^	${SEA}_{45\%}$ (J/g)	$\Delta {SEA}_{45\%}$ (%) ^1^	$F_{45\%}$ (N)	$\Delta F_{45\%}$ (%) ^1^
0.20	654.05	−3.0	0.536	−3.8	759.73	−13.2
0.30	674.38	-	0.557	-	875.69	-
0.40	679.45	+0.8	0.583	+4.7	976.66	+11.5

^1^ Percentage change relative to the nominal case (μ=0.30), $\Delta X=(|X_{\mu }-X_{0.30}|/X_{0.30})\times 100$.

**Table 5:** Repetition level of densification outcomes and late-stage compaction classification of the 12 configurations.

Configuration	α	Rep 1	Rep 2	Rep 3	Classification
SFS	30°	D ^1^	D	D	Progressive densification
	45°	I ^2^	I	D	Case-dependent densification
	60°	I	I	I	Interrupted compaction
	75°	I	I	I	Interrupted compaction
SSC	30°	D	D	D	Progressive densification
	45°	D	I	D	Case-dependent densification
	60°	I	I	I	Interrupted compaction
	75°	I	I	I	Interrupted compaction
SDC	30°	D	D	D	Progressive densification
	45°	D	D	D	Progressive densification
	60°	I	I	I	Interrupted compaction
	75°	I	I	I	Interrupted compaction

^1^ D: Progressive densification observed. ^2^ I: Compaction interrupted before coherent densification, associated with cell loss/ejection, row misalignment, or disruption of the intercellular load path.

**Table 6:** Spearman correlation coefficients between the geometric engagement descriptors and mechanical response metrics.

Response Metric	Correlation with $\Lambda_{A}$	Correlation with $\Lambda_{g}$
Mass-normalized initial stiffness	0.067	0.413
Mass-normalized peak force	0.259	0.056
SEA at 45% strain	0.704	−0.224
CFE at 45% strain	0.294	−0.140

**Table 7:** Quantitative comparison between experimental and FE responses for the representative configurations shown in Figure 12.

Geometry	Initial Stiffness $k_{0}$			Peak Force $F_{peak}$			SEA at 45% Strain			Displacement of Late-Stage Force Increase		
	Exp.	FE	Diff. ^1^	Exp.	FE	Diff. ^1^	Exp.	FEA	Diff. ^1^	Exp.	FE	Diff. ^1^
	N/mm	N/mm	%	N	N	%	J/g	J/g	%	mm	mm	%
SDC 30°	115.54±19.20	132.92	15.0	718.99±63.72	659.13	8.3	0.543±0.048	0.600	10.5	40.56±1.77	34.12	15.9
SDC 45°	138.59±34.59	122.38	11.7	821.38±68.28	674.38	17.9	0.570±0.033	0.557	2.1	42.49±3.47	48.13	13.3
SSC 60°	110.53±36.35	75.81	31.4	708.28±143.66	480.48	32.2	0.428±0.055	0.429	0.3	NSR ^2^	NSR ^2^	_
SFS 75°	104.77±25.72	80.04	23.6	563.27±64.39	427.36	24.1	0.361±0.013	0.326	9.6	NSR ^2^	NSR ^2^	_

^1^ Difference. ^2^ No sustained rise.

## Data Availability

The original contributions presented in this study are included in the article. Further inquiries can be directed to the corresponding author.
